# Identifying ventricular tachycardia exit site utilising a coronary sinus lead pace map: a case report

**DOI:** 10.1093/ehjcr/ytae320

**Published:** 2024-07-02

**Authors:** James Mannion, Faizan Rathore, Nicola Hutchison, Jonathan Lyne

**Affiliations:** Electrophysiology Department, Beacon Hospital, Sandyford, Dublin D18 AK68, Ireland; Midlands Regional Hospital Mullingar, Mullingar, Westmeath N91 NA43, Ireland; Electrophysiology Department, Beacon Hospital, Sandyford, Dublin D18 AK68, Ireland; Biosense-Webster Inc., Johnson & Johnson, Pinewood, UK; Electrophysiology Department, Beacon Hospital, Sandyford, Dublin D18 AK68, Ireland; School of Medicine, University College Dublin, Dublin, Ireland

A 69-year-old male with ischaemic cardiomyopathy underwent biventricular cardiac resynchronization therapy device insertion, on a background of heart failure with reduced ejection fraction despite optimal guideline directed medical therapy. He also had recurrent ventricular tachycardia (VT) storm (*Figure 1A*), despite two previous endocardial VT ablations and surgical sympathectomy. Other history included diabetes, hypertension, atrial fibrillation, and coronary artery disease.

**Figure 1:**
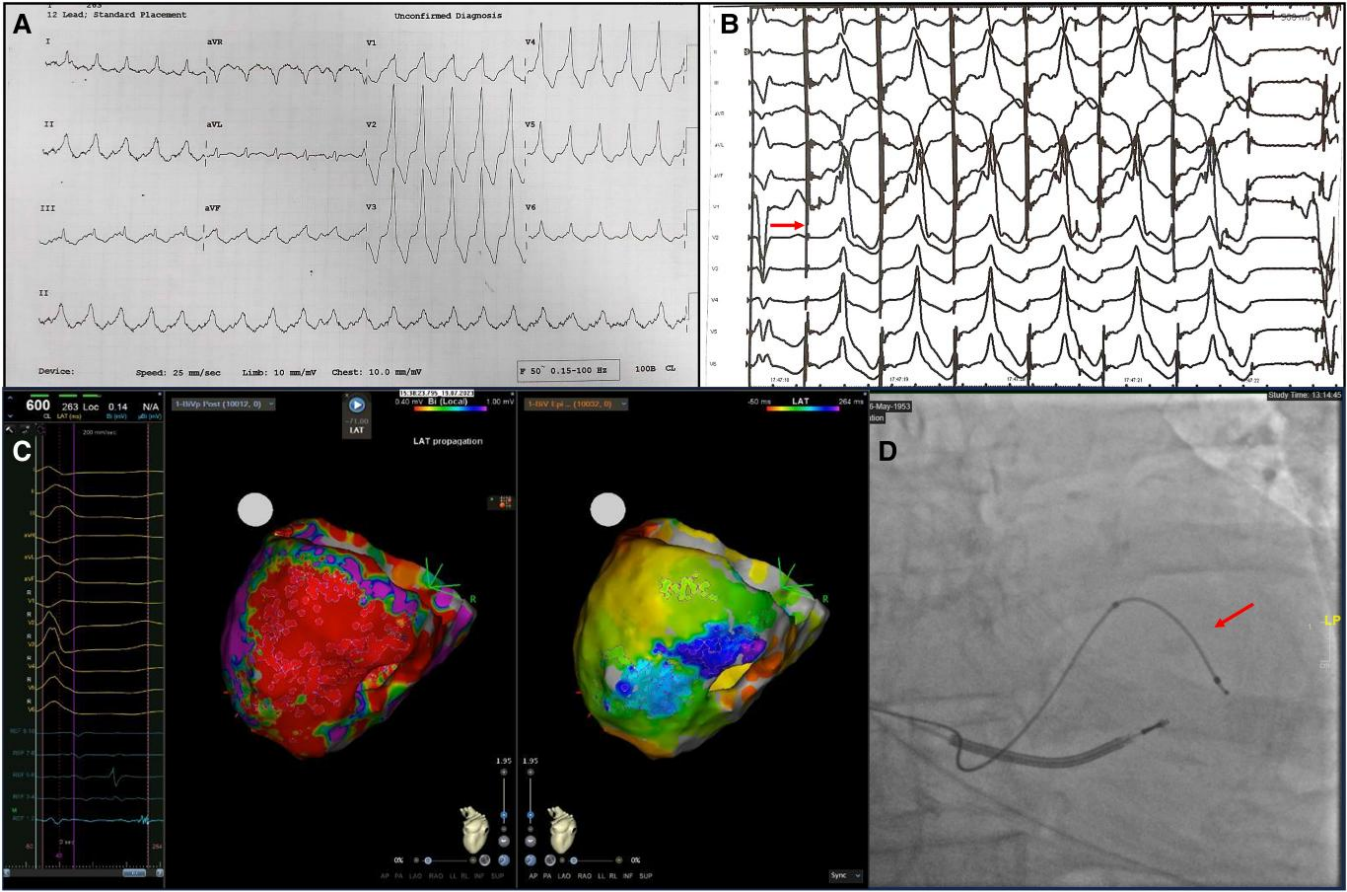
(*A*) Twelve-lead ECG demonstrating recurrent clinical ventricular tachycardia (VT) at a rate of 130 beats per minute. The QRS complexes are very broad, with slurred appearance and right bundle branch block (RBBB) morphology suggesting a left epicardial origin. There is positive concordance across the chest leads, positive in inferior leads II, III, and aVF, positive in lead I, and isoelectric in aVL suggestive of an exit site that is basal, anterior/superior, and lateral. (*B*) Twelve-lead ECG taken at the time of pacing the LV coronary sinus lead. The first complex sequence on the left is an intrinsic left bundle branch block morphology, followed by a pacing spike (arrow) and subsequent change in morphology. There is almost identical QRS morphologies with broad and slurred RBBB appearance, positive concordance across the chest and inferior leads, positive in lead I, and isoelectric in aVL. This is the unipolar paced morphology from the tip electrode of the LV lead located at the mid-antero-lateral wall. (*C*) ECG on left side of panel demarcating latency period of 264 ms. Bipolar voltage map in the centre of the panel with expansive area of low voltage representing scar tissue. On the right side of the panel, we have a local activation time map (LAT) with an area of very late activation identified on the basal lateral wall. This was the epicardial area of ablation. (*D*) Fluoroscopy image of the implantation of the LV coronary sinus lead. Pacing ECG and subsequent local activation time (LAT) map was generated by pacing from the distal electrode in the mid-antero-lateral wall as demonstrated. After further interrogation of the LAT map, ablation targeting areas of late activation was undertaken epicardially at the location identified by the arrow, more basal and inferior than the distal electrode.

During implantation of our left ventricular (LV) coronary sinus lead (ACUITY X4 Quadripolar lead, Boston Scientific), the pacing latency period was noted to be very prolonged (240 ms), with the 12-lead ECG (*Figure 1B*) morphology bearing a strong resemblance to the clinical VT (*Figure 1A*). This was unipolar pacing from the lead tip. It was decided to undertake an epicardial VT ablation approach. Electro-anatomical voltage mapping and local activation time (LAT) mapping were performed with a 10-pole mapping catheter (DecaNavTM, Biosense-Webster Inc.). During paced LAT mapping, an area of very late activation was noted in the epicardial basal infero-lateral wall with a latency period of 264 ms (*Figure 1C*). This territory correlated with the border of a large low voltage area from dense scar. Radiofrequency energy was applied more basal than our LV lead electrode (*Figure 1D*), targeting late potentials. We were unable to induce the VT post-ablation.

Ablation adjacent to device electrodes can cause fibrotic changes at the lead/tissue interface, resulting in impaired sensing, capture, or resynchronization.^1^ Ablation may also lead to structural alterations such as lead insulation compromise, mechanical dislodgements, or coronary sinus stenosis.^1,2^ Lead revisions may be necessary secondary to a rise in device thresholds.^3^

These risks can be mitigated by aiming to keep energy application at least 2 cm away from the device leads or pertinent structures, performing the procedure 6 weeks post-lead insertion and performing checks of device parameters before and after the procedure to ensure preserved function.^1^

## Data Availability

The data underlying this article cannot be shared publicly to maintain the anonymity of the patient involved, in line with the consent obtained.

## Notes

**Consent:** Informed consent to reproduce anonymised information obtained from patient.
